# Transactions of American Medical Association

**Published:** 1877-11

**Authors:** 


### Books Reviewed.

*Transactions of the American Medical Association. Prize Essay. Supplement to Volume XXVII. Excision of the larger joints of the Extremities.* By H. Culbertson, M. D., Professor of Ophthalmology in the Columbus Medical College; Assistant Surgeon U. S. Army, Retired; etc.

Statistics of three thousand nine hundred and eighty cases of excisions of the hip, knee, ankle, shoulder, elbow and wrist, are here presented in a volume of six hundred and seventy-two pages. The collection, tabulation, and analysis of these cases has involved a vast amount of labor, and the work is a very desirable one for the profession to have had performed. The facts presented are from wisely selected cases, and the deductions are carefully drawn. The sections upon the method of operation are very well considered, are accompanied by anatomical illustrations: this portion adds much to the value of the work. The faithfulness, intelligence and accuracy shown in the compilation of these cases reflects much credit upon the author.

The chief value of the work is found more in the presentation it makes of the particular facts in many different cases, than in the practical indications of the analysis. The general experience of the profession is not sufficient yet to indicate properly the eligibility of excision in various points. It is an excellent thing to have the work brought before the profession, as it will incite more general trial of these operations, and it serves as a nucleus for the gathering of further cases in evidence. The number of cases reported in this work by American operators is twice that of English or German surgeons. The knee-joint is shown to have been excised in England twice as often for disease as in the United States or Germany; the mortality being the least in the United States. The mortality of excision of the knee from the several causes in general varies widely. Serviceability of limb appears in fifty-six per cent. of the recoveries from excision of the knee for disease; fifty-eight for gunshot wounds; eighty-two for injuries, and eighty-seven for deformity. Beside the knee, England shows the largest number of excisions of the ankle. United States has the highest number of excisions of the shoulder, elbow, hip and wrist. Germany presents a much larger number of excisions of shoulder, elbow, and wrist than England. France furnishes a much smaller number of excisions than the three before mentioned countries. It is not to be supposed however, that other countries are equally well represented with United States by our author.

The case of Mr. Cash, seventy-six years of age, first reported by Professor J. F. Miner, in volume three of the JOURNAL, of immediate extraction of the head of the femur fractured and displaced by injury, with recovery, is specially mentioned and unique, no other case being reported of immediate removal, though twenty-two are given of removal after separation by morbus coxarius, and one several months after an injury causing fracture and chronic arthritis.

It will require more time for the profession to be settled in opinion on the subject of excision of joints. Various authors speak each for themselves and much more is to be depended upon the individual judgment and experience of writers than upon general conclusions from statistics at present obtainable.

